# A practical approach to assess leg muscle oxygenation during ramp-incremental cycle ergometry in heart failure

**DOI:** 10.1590/1414-431X20176327

**Published:** 2017-10-02

**Authors:** A.C. Barroco, P.A. Sperandio, M. Reis, D.R. Almeida, J.A. Neder

**Affiliations:** 1 Disciplina de Cardiologia, Universidade Federal de São Paulo, São Paulo, SP, Brasil; 2 Departamento de Cardiologia, Instituto Dante Pazzanese de Cardiologia, São Paulo, SP, Brasil; 3 Setor de Fisiologia Clínica do Exercício, Disciplina de Pneumologia, Universidade Federal de São Paulo, São Paulo, SP, Brasil; 4 Departamento de Fisioterapia, Faculdade de Medicina, Universidade Federal do Rio de Janeiro, Rio de Janeiro, RJ, Brasil; 5 Laboratory of Clinical Exercise Physiology, Division of Respiratory and Critical Care Medicine, Queen's University, Kingston, ON, Canada

**Keywords:** Heart failure, Exercise, Oxygen, Muscle, Near-infrared spectroscopy

## Abstract

Heart failure is characterized by the inability of the cardiovascular system to maintain oxygen (O_2_) delivery (i.e., muscle blood flow in non-hypoxemic patients) to meet O_2_ demands. The resulting increase in fractional O_2_ extraction can be non-invasively tracked by deoxygenated hemoglobin concentration (deoxi-Hb) as measured by near-infrared spectroscopy (NIRS). We aimed to establish a simplified approach to extract deoxi-Hb-based indices of impaired muscle O_2_ delivery during rapidly-incrementing exercise in heart failure. We continuously probed the right vastus lateralis muscle with continuous-wave NIRS during a ramp-incremental cardiopulmonary exercise test in 10 patients (left ventricular ejection fraction <35%) and 10 age-matched healthy males. Deoxi-Hb is reported as % of total response (onset to peak exercise) in relation to work rate. Patients showed lower maximum exercise capacity and O_2_ uptake-work rate than controls (P<0.05). The deoxi-Hb response profile as a function of work rate was S-shaped in all subjects, i.e., it presented three distinct phases. Increased muscle deoxygenation in patients compared to controls was demonstrated by: i) a steeper mid-exercise deoxi-Hb-work rate slope (2.2±1.3 *vs* 1.0±0.3% peak/W, respectively; P<0.05), and ii) late-exercise increase in deoxi-Hb, which contrasted with stable or decreasing deoxi-Hb in all controls. Steeper deoxi-Hb-work rate slope was associated with lower peak work rate in patients (r=–0.73; P=0.01). This simplified approach to deoxi-Hb interpretation might prove useful in clinical settings to quantify impairments in O_2_ delivery by NIRS during ramp-incremental exercise in individual heart failure patients.

## Introduction

Heart failure is a complex syndrome characterized by the inability of the cardiovascular system to maintain oxygen (O_2_) delivery [i.e., muscle blood flow (Q̇_m_)] matched to metabolic demands (1). This is particularly true during dynamic exercise as the peripheral muscle requirements for O_2_ increase markedly (2,3). In fact, there is well-established evidence that the deleterious bioenergetic consequences (e.g., early anaerobic metabolism) of impaired O_2_ availability are centrally related to patients' exercise intolerance (4). Selected pharmacological (e.g., sildenafil intake) and non-pharmacological therapies (i.e., physical training) have been found useful in improving Q̇_m_-O_2_ uptake (V̇O_2_) matching with important beneficial consequences to patients functioning (5–7).

In this context, there is a widespread interest in non-invasive methods to detect impairments in exercise Q̇_m_-V̇O_2_ matching in heart failure patients (5). Near infrared spectroscopy (NIRS), in particular, is an optical method that allows transcutaneous monitoring of skeletal muscle deoxygenation (deoxi-Hb), an index of fractional O_2_ extraction (8,9). It has been postulated that muscle deoxi-Hb can reflect dynamic abnormalities in Q̇_m_-V̇O_2_ coupling when the rate of increase in V̇O_2_ is constant, e.g., in response to a rapidly-incremental (ramp) exercise protocol. Thus, higher values and/or faster increases in deoxi-Hb would result from insufficient Q̇_m_ relative to V̇O_2_ as muscle O_2_ extraction increases to compensate for insufficient O_2_ delivery (4,10). Despite its potential clinical usefulness, this approach has been mostly used in healthy subjects (4). Moreover, the response profile has been described by complex non-linear mathematical models (either the hyperbolic or sigmoid functions) (4,10). As pointed out by Spencer et al. (11), fitting the whole response in a single function has little physiological rationale and it might represent a “fit of convenience”. Translating the deoxi-Hb signal to the clinical world using a practical and feasible approach remains an important gap to allow a wider use of NIRS for the functional assessment of heart failure patients.

This prospective study was designed to establish a novel, clinically-friendly approach to quantify Q̇_m_-V̇O_2_ mismatch by deoxi-Hb during ramp-incremental exercise in patients with heart failure. We specifically hypothesized that impairments in peripheral muscle O_2_ delivery would be indicated by steeper mid-exercise deoxi-Hb-work rate slope and/or greater increases in late-exercise deoxi-Hb in patients compared to healthy controls.

## Subjects and Methods

### Subjects

Ten non-smoking males from the heart failure outpatient clinic of the São Paulo Hospital (New York Heart Association functional score II and III) and 10 age- and gender-matched healthy controls were assessed. Patients presented with left ventricular ejection fraction (LVEF) <35% according to 3-D transthoracic echocardiogram. They were under optimal pharmacological treatment for stage “C” patients as established by the American Heart Association and American College of Cardiology guidelines (2). We excluded patients who had a history of recent disease decompensation (within 3 months), functional evidence of obstructive pulmonary disease (forced expiratory volume in 1 s/forced vital capacity <0.7), anemia (hemoglobin <13 g/dL), exercise-induced asthma, diabetes mellitus or other metabolic disease, significant ventricular arrhythmia, atrial fibrillation, unstable angina, acute myocardial infarction in the preceding year, and peripheral arterial disease associated with intermittent claudication. No patient had been previously submitted to cardiovascular rehabilitation to avoid the influence of physical activity on muscle oxygenation (12).

Controls were office staff and non-medical employees from the Universidade Federal de São Paulo. They were required to be sedentary as indicated by lack of regular physical activity in the preceding 5 years. No control presented with a previous history of pulmonary, cardiovascular, autoimmune or metabolic diseases. Prior to study inclusion, the controls underwent clinical assessment, pulmonary function tests, blood analysis (including Hb) and resting electrocardiogram and echocardiogram. The study protocol was approved by the Institutional Research Ethics Board and all participants gave written informed consent (Project #0935/07).

### Measurements

#### Cardiopulmonary exercise test

Cycle ergometer-based (Corival® 400, Medical Graphics Corporation, MGC, USA) cardiopulmonary exercise test (CardiO_2_ system, MGC) was performed following a ramp-incremental protocol (5–10 W/min for patients and 5–20 W/min for controls). Subjects were asked to cycle at a frequency of 50±5 rpm. Peak V̇O_2_ (mL/min) was the highest value obtained at exercise cessation: values were compared with those predicted by Neder and co-workers (13). Other measurements included: CO_2_ output (V̇CO_2_, mL/min), R (respiratory exchange ratio), minute ventilation (V̇E, L/min), respiratory rate (*f*), ventilatory equivalents for O_2_ and CO_2_ (V̇E/V̇O_2_ e V̇E/V̇CO_2_) and end-tidal partial pressure of O_2_ and CO_2_ (P_ET_O_2_ e P_ET_CO_2_, mmHg). Heart rate (HR, bpm) was determined using R-R distance as determined by a 12-lead electrocardiogram (CardioPerfect™, MGC). Oxygen saturation was determined by pulse oximetry (SpO_2_, Onyx™, Nonim, USA). Patients were asked about their dyspnea and leg effort every 2 min according to the 0-10 Borg scale. The V̇O_2_ at the lactate threshold was estimated by the gas exchange method (modified *V-*slope) and confirmed by the ventilatory method, i.e., V̇E/V̇O_2_ and P_ET_O_2_ increase coupled with V̇E/V̇CO_2_ and P_ET_CO_2_ stability.

### Peripheral muscle oxygenation

Leg muscle deoxygenation was measured by the NIRO 200® system (Hamamatsu Photonics, Japan). The NIRS theory has been described elsewhere (9). Briefly, an optical fiber bundle carries the near-infrared light produced by a laser diode to the tissue while another optical fiber bundle captures the tissue-transmitted light to a photon detector in the spectrometer. The light intensity and the transmitted light is continuously recorded and, along with the relevant extinction coefficient, used to measure changes in the hemoglobin oxygenation level (Hb) and myoglobin (Mb). The optodes (light emitting and photoreceptor sensors) were set at the vastus lateralis muscle of the left quadriceps, between the lateral epicondyle and greater trochanter of the femur, fixed with an appropriate adhesive tape and covered with a neoprene band to avoid light penetration.

The variables evaluated by NIRS were oxygenated and deoxygenated Hb concentrations (oxi-Hb and deoxi-Hb, respectively). From these primary signals, total Hb is derived, i.e., oxi-Hb + deoxi-Hb. Considering that about 70% of the Hb intramuscular signal comes from venous bed, variations in local blood volume (including venous) are expected to impact more oxi-Hb than deoxi-Hb (14–16). Thus, many laboratories have adopted deoxi-Hb as the preferred marker for changes in the O_2_ fractional extraction (14,15,17), i.e., an index of Q̇m-V̇O_2_ (mis)match (18). The device used here (continuous wave NIRS) does not measure light tissue reflection and scattering (18); thus, values were recorded as a variation (Δ) from baseline in mMol/cm and are reported as a percent of the end-test value, i.e., 0–100%.

### Statistical analysis

The statistical program used was SPSS® version 13.0 (SPSS®, USA). Unless otherwise stated, data are reported as means and SD. Unpaired *t*-test (or Mann-Whitney test, when appropriated) was used for between-group comparisons. The slope of linear regression involving exercise deoxi-Hb as a function of work rate determined the rate of increase in the former variable. Pearson correlation was used to assess the level of linear association between continuous variables. The level of statistical significance was set at <5% (P<0.05) for all tests.

## Results

There were no significant between-group differences in anthropometric attributes (Table 1). The main etiology of heart failure was non-ischemic cardiomyopathy and, as expected by the inclusion criteria, all patients showed severe left ventricular dysfunction. Peak work rate and peak V̇O_2_ were markedly reduced in patients; for instance, 7 patients were on Weber’s class C. Patients had shallower V̇O_2_-work rate slopes than controls; conversely, V̇E/V̇CO_2_ was higher and P_ET_CO_2_ lower in patients compared to controls (P<0.05; Table 1).

**Table 1.** Resting and exercise characteristics of healthy controls and patients with heart failure.

Variables	Controls (n=10)	Heart failure (n=10)
Demographic/anthropometric		
Age (year)	61.5±9.3	52.1±11.7
Weight (kg)	76.5±9.1	72.0±16.4
Height (cm)	168.7±5.3	166.7±8.6
Body mass index (kg/m^2^)	27.0 ±3.0	25.8±4.9
Echocardiogram		
Left ventricular ejection fraction (%)	59.7±18.7	29.1±4.9*
Medication		
Thiazide diuretics (N)	–	7
Spironolactone (N)	–	4
Digitalis (N)	–	5
Carvedilol (N)	–	10
ACE Inhibitors/ AR blockers (N)	–	10
Incremental exercise		
Peak work rate (W)	141±28	80±26*
Peak V̇O_2_ (mL/min)	1758±313	1134±416*
Peak V̇O_2_ (mL·min^-1^·kg^-1^)	23.1±3.8	15.4±4.9*
V̇O_2_LT (mL/min)	746±120	634±153
V̇O_2_-work rate slope (mL·min^-1^·W^-1^)	10.5±0.8	8.8±1.7*
Peak RER	1.21±0.09	1.04±0.16*
Peak V̇E/V̇CO_2_	34.3±5.4	47.6±13.5*
Peak P_ET_CO_2_ (mmHg)	35.0±5.2	27.1±10.5
Peak HR (bpm)	140±26	131±15

Data are reported as means ± SD or frequency (N). ACE: angiotensin-converting enzyme; AR: angiotensin receptor; V̇O_2_: oxygen uptake; LT: lactate threshold; RER: gas exchange ratio; V̇E: ventilation; V̇CO_2_: carbon dioxide output; P_ET_: end-tidal partial pressure; HR heart rate.

* P<0.05 (unpaired *t*-test).

As previously described in normal subjects (4,10), deoxi-Hb response curve as a function of increasing work rate was S-shaped i.e., it resembled a sigmoid in all subjects. From the raw signal, we initially identified two inflection points: point “A” corresponded to the work rate after exercise onset at which deoxi-Hb started to systematically increase, and from point “A” onward we applied linear regression to deoxi-Hb. Point “B” corresponded to the work rate at which there was a systematic departure from linearity.

**Figure 1.**
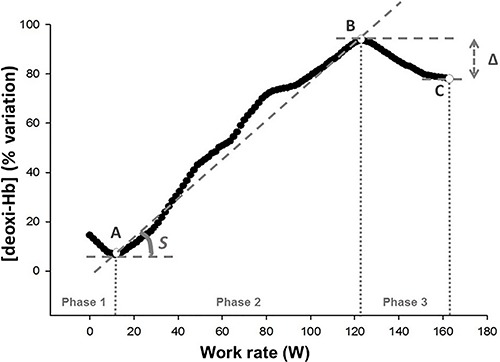
Representative deoxygenated hemoglobin concentration (deoxi-Hb) response profile (% rest-peak variation) as a function of increasing exercise intensity in a healthy control. Points “A” and “B” correspond to the first and second inflection points. Point “C” is the peak work rate. In addition to the slope (*S*) of deoxi-Hb increase throughout phase “2”, deoxi-Hb difference between points “B” and “C” is depicted (“Δ”).

The range of work rates before point “A”, between points “A” and “B” and after point “B” up to peak exercise (point “C”) were named phases “1”, “2” and “3”, respectively. In addition to the increase in slope (*S*) of deoxi-Hb throughout phase 2, we calculated the deoxi-Hb difference (“Δ”) between points “B” and “C” (Figure 1).

**Figure 2.**
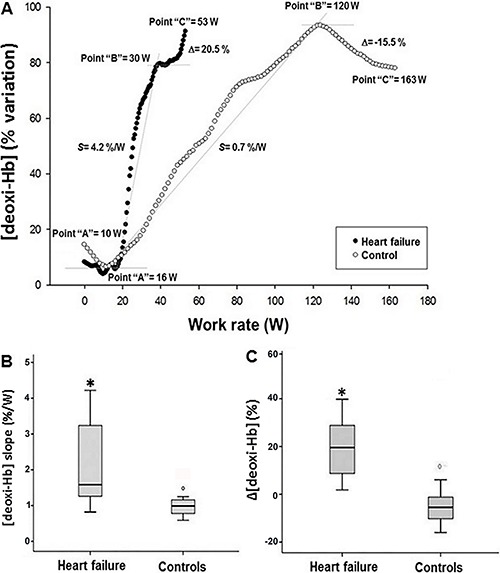
Representative deoxygenated hemoglobin concentration (deoxi-Hb) response profile (% rest-peak variation) as a function of increasing exercise intensity in a representative control and a patient with heart failure (*panel A*). Lower panels show box plots comparing the slope of deoxi-Hb increase as a function of work rate throughout phase “2” (*panel B*) and Δdeoxi-Hb difference between points “B” and “C” (*panel C*). Data are reported as means±SD. Variables: [deoxi-Hb] Slope (S): Slope of the ratio [deoxi-Hb]/work-rate (%variation/W); Δ[deoxi-Hb] (Δ): variation of [deoxi-Hb] at the maximum work-rate point (C) to the second inflection point (B). *P<0.05: Unpaired *t*-test (*panel B*) and Mann-Whitney test (*panel C*).

As shown in Figure 2 for representative subjects and in Table 2 and Figure 2 for mean data, patients presented with significant steeper deoxi-Hb slope than controls (P<0.01). Moreover, while deoxi-Hb remained stable or even decreased during phase “3” in all but one control (i.e., null or negative “Δ”), there were systematic increases in deoxi-Hb in all patients (P<0.05). Steeper deoxi-Hb-work rate slope was associated with lower peak work rate in patients (r=–0.73; P=0.01).

**Table 2.** Key variables of deoxygenated hemoglobin concentration (deoxi-Hb)-work rate relationship in healthy controls and patients with heart failure.

Variables	Controls (n=10)	Heart failure (n=10)
Point “A” (W)	19±14	18±11
Point “B” (W)	111±32	53±19*
[deoxy-Hb] slope (%/W)	1.0±0.3	2.2±1.3*
Δ[deoxy-Hb] (%)	-0.5±18.9	20.3±12.9*

Point “A”: work rate after exercise onset at which deoxi-Hb started to increase; Point “B”: work rate at which there was a systematic departure from linearity; [deoxi-Hb] Slope: slope of the ratio [deoxi-Hb]/work-rate (%variation/W); Δ[deoxi-Hb]: variation of [deoxi-Hb] at the maximum work-rate point (C) to the second inflection point (B). Data are reported as mean±SD.

* P<0.05: unpaired *t-*test, except “Δ” (Mann-Whitney test).

## Discussion

This prospective study established a simplified approach to unravel abnormalities in peripheral muscle O_2_ delivery (i.e., lower blood flow in non-hypoxemic patients) as indicated by changes in NIRS-based deoxi-Hb during ramp-incremental cardiopulmonary exercise test in heart failure patients. Our results indicate that, compared to controls, patients presented with steeper mid-exercise slope of deoxi-Hb as a function of work rate coupled with lack of late-exercise stability (or even decreasing deoxi-Hb). We interpret these results as evidence of faster and higher O_2_ extraction to compensate for impaired convective and diffusive O_2_ flow to muscle mitochondria (10). This approach might prove useful to assess the effects of pharmacological and non-pharmacological methods aimed at improving intra-muscular microvascular hemodynamics in this patient population. The proposed approach can be easily applied in clinical settings, as it does not require data fitting with complex mathematical functions (7). Moreover, deoxi-Hb is reported as a function of work rate, and V̇O_2_ measurements (i.e., cardiopulmonary exercise test) are not mandatory. Importantly, the proposed parameters (slope and “Δ”) are largely effort-independent, being recorded during submaximal exercise.

From a mechanistic standpoint, it has been long established that the key factors modulating O_2_ delivery-utilization matching in contracting appendicular muscles include: a) the muscle “pump” effect; b) local vasodilatation; c) parasympathetic and sympathetic tones, and d) differential patterns of muscle fiber recruitment, as reviewed by other authors (4,10,19). Based on these premises, we interpreted the S-shaped pattern of muscle deoxygenation (deoxi-Hb) depicted in Figure 1 as indicating: a) an early phase (“1”) in which proportional increases in O_2_ delivery and O_2_ requirements (V̇O_2_) led to a stable O_2_ extraction (∼deoxi-Hb), b) a subsequent phase (“2”) in which deficits in O_2_ delivery relative to fast-increasing V̇O_2_ produced a marked increase in O_2_ extraction, and c) a final phase (“3”) in which O_2_ delivery and O_2_ requirements were once again matched leading to a stable rate of O_2_ extraction (or even decreasing if O_2_ delivery becomes excessive relative to instantaneous O_2_ needs) (4,10). This model is consistent with previous contentions by Spencer et al (11), who found that the deoxi-Hb response profile during ramp-incremental exercise in healthy young males consisted of three distinct phases, in which the latter two were approximately linear, i.e., phases “1” and “2” herein described.

In this context, steeper phase “2” deoxi-Hb-work rate slope in patients than controls is strongly suggestive of impaired O_2_ delivery-utilization matching in the former group. It is noteworthy that these abnormalities occurred despite a shallower V̇O_2_-work rate slope in patients. Thus, even if changes in O_2_ requirements were lower in patients, marked deficits in Q̇_m_ likely precluded a corresponding increase in O_2_ delivery. In other words, V̇O_2_/extraction ratio was markedly reduced in patients, a finding consistent with impaired O_2_ delivery. Increased O_2_ extraction in patients might have also been influenced by lactacidosis-induced rightward shifts in the oxy-hemoglobin dissociation curve (Bohr effect) and/or greater recruitment of O_2_-costly type II fibers. Thus, a direct quantitative (inverse) relationship between Q̇_m_ and deoxi-Hb should not be attempted.

Progressive increase in late-exercise (phase “3”) deoxi-Hb in patients, but not in controls, is another evidence of poorer muscle O_2_ delivery-utilization matching in heart failure conditions. In fact, there is growing evidence that despite progressive increases in work rate (and V̇O_2_), cardiac output might stabilize (or even decrease) near peak exercise in these patients (20–22). Microvascular perfusion-muscle fiber recruitment uncoupling (4,10,21) and sympathetic over-excitation (23) may also further impair Q̇_m_ near exercise termination. Moreover, type II fibers (with lower ATP/O_2_ ratio) are mostly recruited at higher compared to lower work rates (24,25), which might have contributed to muscle O_2_ delivery- V̇O_2_ mismatch in phase “3”.

As a noninvasive, cross-sectional study our investigation has some limitations that should be highlighted. We assume, as others (5,8,26–28), that deoxi-Hb reflects muscle fractional O_2_ extraction (C(a-v)O_2_); however, we did not measure blood gas tensions. We also assumed that deoxi-Hb at a specific site gives a rough estimate of overall muscle O_2_ extraction (8,14,16,28). Koga et al. (18), however, found large heterogeneity in Q̇_m_-V̇O_2_ distribution in normal subjects, a phenomenon that might be more relevant for poorly perfused muscles. There is mounting evidence that Q̇_m_-V̇O_2_ distribution abnormalities worsen as disease progresses in humans (29,30) and animals (31,32). Thus, our approach needs to be tested in more impaired patients. Finally, patients performed a cycle ergometer test as the NIRS signal quickly deteriorates during fast walking; thus, our approach is unlikely to be feasible for treadmill-based tests.

In conclusion, we presented a practical approach to interpret the deoxi-Hb signal by NIRS during ramp-incremental cycle ergometry in heart failure patients. Impairments in O_2_ delivery, likely reflective of poor muscle blood flow in non-hypoxemic patients, were non-invasively uncovered by steeper mid-exercise slope of deoxi-Hb as a function of work rate and increasing (instead of stable or decreasing) deoxi-Hb near peak exercise. This novel strategy might prove useful to assess the effects of pharmacological and non-pharmacological interventions aimed at improving skeletal muscle perfusion in this patient population.
